# Drug resistance reversal in ovarian cancer cells of paclitaxel and borneol combination therapy mediated by PEG-PAMAM nanoparticles

**DOI:** 10.18632/oncotarget.19728

**Published:** 2017-07-31

**Authors:** Liang Zou, Di Wang, Yichen Hu, Chaomei Fu, Wei Li, Liping Dai, Lin Yang, Jinming Zhang

**Affiliations:** ^1^ School of Medicine, Chengdu University, Chengdu 610106, China; ^2^ College of Pharmacy and Biological Engineering, Chengdu University, Chengdu 610106, China; ^3^ College of Pharmacy, Chengdu University of Traditional Chinese Medicine, Chengdu 611137, China

**Keywords:** MDR reversal, co-delivery, P-gp inhibition, PAMAM dendrimer, borneol

## Abstract

Paclitaxel (PTX) is frequently suffered from multidrug resistance (MDR), resulting in lower chemotherapeutic efficacy and even chemotherapy failure. To combine the P-glycolprotein (P-gp) inhibitor would be a useful strategy to overcome MDR. However, what is needed now is an efficient vehicle to deliver multiple drugs into tumor simultaneously. In this study, PTX and Borneol (BNL), a natural compound with P-gp inhibition effect confirmed in intestinal absorption, were co-loaded in the fabricated PEG-PAMAM nanoparticle (NPs) by a one-step nano-precipitation method with high drug loading efficiency, narrow size distribution and low hemolysis rate. Based on P-gp inhibition activity of BNL, confirmed by drug efflux test and molecular docking model, the combination of PTX and BNL could improve intracellular concentration of PTX in A2780/PTX cells. Furthermore, compared to both free PTX and PTX+BNL, PB/NPs and P/NPs plus BNL exhibited higher cellular uptake and cytotoxicity in A2780/PTX cells, as well as the decreased MMP and enhanced apoptosis rate. More importantly, although PB/NPs and P/NPs+B showed similar tumor accumulation in tumor-bearing mice, PB/NPs could significantly decrease tumor growth of A2780/PTX tumor-bearing mice, in comparison to P/NPs+B. These results indicated the advantage of PTX and BNL co-delivery NPs for MDR reversal. These findings demonstrate that the co-delivery nano-sized system comprised by PEG-PAMAM polymer with PTX and BNL co-loaded would be a promising candidate for MDR treatment.

## INTRODUCTION

Ovarian cancer is the fifth most common gyneco-logic malignancy in women and the leading cause of deaths in developed countries [1]. Paclitaxel (PTX), also called taxol, has been adapted as one of the standard agents for ovarian cancer treatment [2]. However, more than 70% of ovarian cancer patients with the initial PTX chemotherapy eventually suffer from multidrug resistance (MDR), which is the major obstacle for ideal chemotherapeutic outcome [3]. Multiple mechanisms, including the increased drug efflux, decreased drug intake, activation of detoxifying systems, activation of DNA repair process, and the evasion of drug-induced apoptosis, have been found to associate drug-resistance [4, 5]. Thereinto, the overexpression of the most commonly efflux membrane transporter P-glycoprotein (P-gp) is a fatal factor, to pump out drugs out of cancer cells [6]. Previous researches have mainly focused on an essential strategy to reverse drug-resistance by a combination of small molecular compound inhibitors, such as verapamil, quinidine, progesterone, tamoxifen, phenothiazines and so on, to inhibit P-gp expression [7–9]. Borneol (BNL), a Chinese materia medica monomer (molecular weight 154.24), is extracted from *Dryobalanops aromatica Gaertn f*. and *Blumea balsamifera DC*, or chemically synthesized from camphor and turpentine oil. Previous studies reported that BNL could improve the intestinal absorption [10] and blood brain barrier permeability [11] of some drugs, owing to its P-gp suppression effect. Similarly, BNL was also demonstrated to improve anticancer efficacy by means of enhancing cellular uptake related to P-gp suppression [12]. However, to the best of our knowledge, the co-delivery of BNL with a cytotoxic agent has not been described in the literature as an approach to overcome MDR.

Unfortunately, the drug-resistance reversal effect of combination therapy is often limited by the different pharmacokinetics of multiple drugs, thereby resulting from the uncoordinated cellular uptake of various drugs in tumor cells and reducing their synergistic anticancer effects. The co-delivery of dual or multiple agents via a single nanocarrier is a promising approach to further improve the combination therapy and overcome the limits abovementioned. Many types of nano-constructs have been employed as the drug delivery vehicles, such as polymeric micelles, lipid-related nanoparticles, dendrimers, and various inorganic nanoparticles. Some previous studies on co-delivery of PTX and lonidamine [13], as well as PTX and resveratrol co-encapsulation in liposomes [14], provided representative illustrations for MDR reversal by means of co-delivering cytotoxic drug and MDR modulator in a single nano-vehicle.

Among these, PAMAM dendrimers are one kind of ideal drug delivery system, with hyper-branched, nano-sized and well-defined architecture [15–17]. Specifically, PAMAM dendrimers could enhance water solubility and bioavailability of poorly water-soluble drug, protect drugs from premature elimination and exhibit high drug loading efficiency via either covalent conjugation [18, 19] or physical loading in interior cavity [20, 21]. However, the significant drawbacks of PAMAM on its cytotoxicity and rapid elimination in systemic circulation, due to the exposure of positive amine groups on the surface, greatly limit its application. Commonly, PEGylation modification [22, 23] is the effective approach to neutralize the positive charged and improve its biocompatibility. In this study, PTX and BNL were co-loaded in PEG-PAMAM nanoparticles (NPs) (PB/NPs) and evaluated the MDR reversal of paclitaxel-resistant ovarian cancer A2780/PTX cells *in vitro* and *in vivo*, compared with free combination of PTX and BNL (P+B), as well as the combination of PTX NPs and free BNL (P/NPs+B). We also investigated the enhanced cellular uptake *in vitro* and tumor tissue accumulation *in vivo*. The essential aim of this study is to reveal the potential role for MDR treatment reversal mediated the combination of BNL and nano-carriers.

## RESULTS AND DISUSSION

### Characterization of PEG-PAMAM NPs

**Figure 1:**
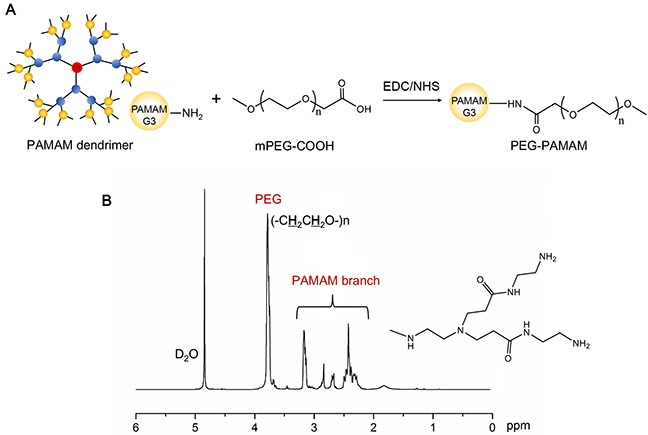
Synthesis scheme **(A)** and ^1^H NMR spectra **(B)** of PEG-PAMAM polymer.

The synthesis scheme of PEG-PAMAM polymer was displayed in Figure 1A. Initially, PEG-PAMAM polymers were synthesized by the conjugation between carboxyl-terminal of mPEG and amino-terminal of PAMAM dendrimer via the amidation reaction through EDC/NHS catalytic action. During this reaction, carboxyl groups of mPEG-COOH were activated and converted to NHS ester, and then coupled with amino groups on PAMAM dendrimer surface. The successful synthesis could be demonstrated by ^1^H NMR. As shown in Figure 1B, the chemical shift of PEG (-C**H**_2_C**H**_2_O-) was found at 3.72 (ppm, peak a). Some representative chemical shifts derived from PAMAM branch could confirm the PAMAM dendrimer, such as δ PAMAM (-CONHC**H**_2_-) at 3.26 (ppm, peak b), δ PAMAM (-C**H**_2_CONH-) at 2.39 (ppm, peak c), and a series of peaks at 2.6∼3.0 (ppm, peak d) from protons next to amines.

**Figure 2:**
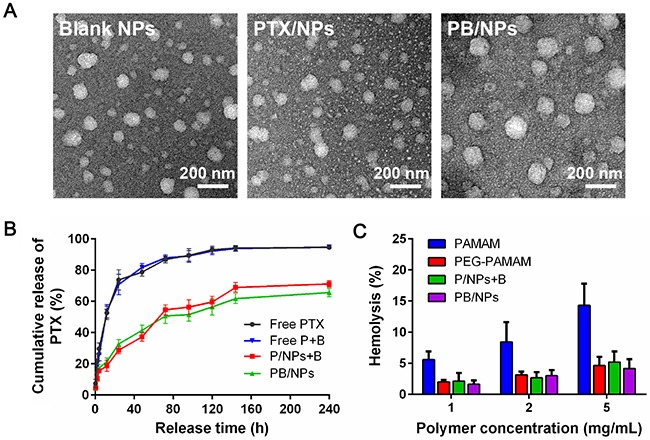
Characterization of PEG-PAMAM NPs **(A)** TEM images of blank NPs, PTX/NPs and PTX+BNL co-delivery NPs; **(B)** Drug release profiles of PTX from NPs; **(C)** Hemolysis rate of various PAMAM-derivative formulations.

Based on the nanoprecipitation method, liposoluble drugs could be loaded in hydrophobe core of PAMAM dendrimer. As shown in Table 1, PEG-PAMAM NPs possess suitable average size lower than 100 nm and narrow size distribution, in which PDI was lower than 0.2. Especially, though zeta potential of PEG-PAMAM NPs exhibited slightly positive, PEG coating on the surface of PAMAM dendrimer still dramatically reduced zeta potential of PAMAM dendrimer, due to the a mass of amino groups on PAMAM surface. PEGylation on PAMAM resulted in the dropped positive zeta potential, which would accordingly increase stability in storage and reduce the hemolysis in blood circulation. PTX could be loaded in PEG-PAMAM NPs with high encapsulation efficiency of 89.6% and 84.7% in single PTX/NPs and PTX+BNL co-loaded NPs (PB/NPs). The morphology images in Figure 2 shown that singe PTX/NPs and PB/NPs exhibited spherical shape. It should be noted that because of the abundant nanocavities of PAMAM, PTX in this nano-carrier exhibited improved encapsulation efficiency, compared to liposomes and PEG-PLGA micelles.

**Table 1:** Physicochemical characteristics of single PTX/NPs and PTX+BNL co-loaded NPs

Formulation	Size (nm)	PDI	Zeta potential (mV)	EE (%)		LE (%)	
				PTX	BNL	PTX	BNL
Blank NPs	86.6±4.5	0.14±0.01	4.4±0.6	-	-	-	-
P/NPs	92.1±5.2	0.18±0.02	4.9±0.3	89.6	-	3.42	-
PB/NPs	91.8±5.0	0.13±0.01	3.3±0.2	84.7	96.4	2.86	10.42

The *in vitro* release of PTX from free drug state and nanovehicles were evaluated in PBS (pH 7.4). As shown in Figure 2, PTX in PEG-PAMAM NPs exhibited sustained-release profiles in comparison of rapid release from free drugs. PTX without loading exhibited fast drug burst and almost 90% of PTX could be released from free PTX and PTX+BNL mixture at 96 h. However, PTX loaded in NPs shown the remarkable gradual drug release, in which only 60% of PTX could be escaped at 240 h. The single PTX/NPs and PB/NPs displayed the similar sustained drug release behavior. This should be due to the fact that PTX were well embedded in the hydrophobic cavities of PAMAM dendrimer and surrounded by the outer hydrophilic PEG layer. The slow drug release of PTX in NPs demonstrated that drugs in PEG-PAMAM NPs could keep stable and avoid the premature drug leakage in blood circulation. Additionally, owing to the intravenous injection, the biocompatibility of samples must be evaluated by means of *in vitro* hemolytic test. As shown in Figure 2, PAMAM dendrimers exhibited significant hemolytic activity with the time-dependent manner. The serious hemolysis of PAMAM dendrimers was resulted from the conjugation of abundant amino groups on the surface of PAMAM with the anions on the membrane of erythrocytes, and subsequently cause the hemoglobin release into plasma. Thus, PEGylation on PAMAM remarkably reduced the hemolysis rate, for the reason of the neutralization of positive potential. Even 5 mg/mL of PEG-PAMAM polymers only resulted in less than 5% hemolysis rate. So, accordingly both PTX/NPs and PB/NPs composed by PEG-PAMAM polymer showed the low hemolysis rate as similar with PEG-PAMAM polymers. The lower than 5% of hemolysis rate of NPs could be regarded safe for intravenous administration [24], indicated that PEG-PAMAM NPs possessed good blood compatibility and enough safety for clinical application.

### Cellular uptake of PEG-PAMAM NPs

**Figure 3:**
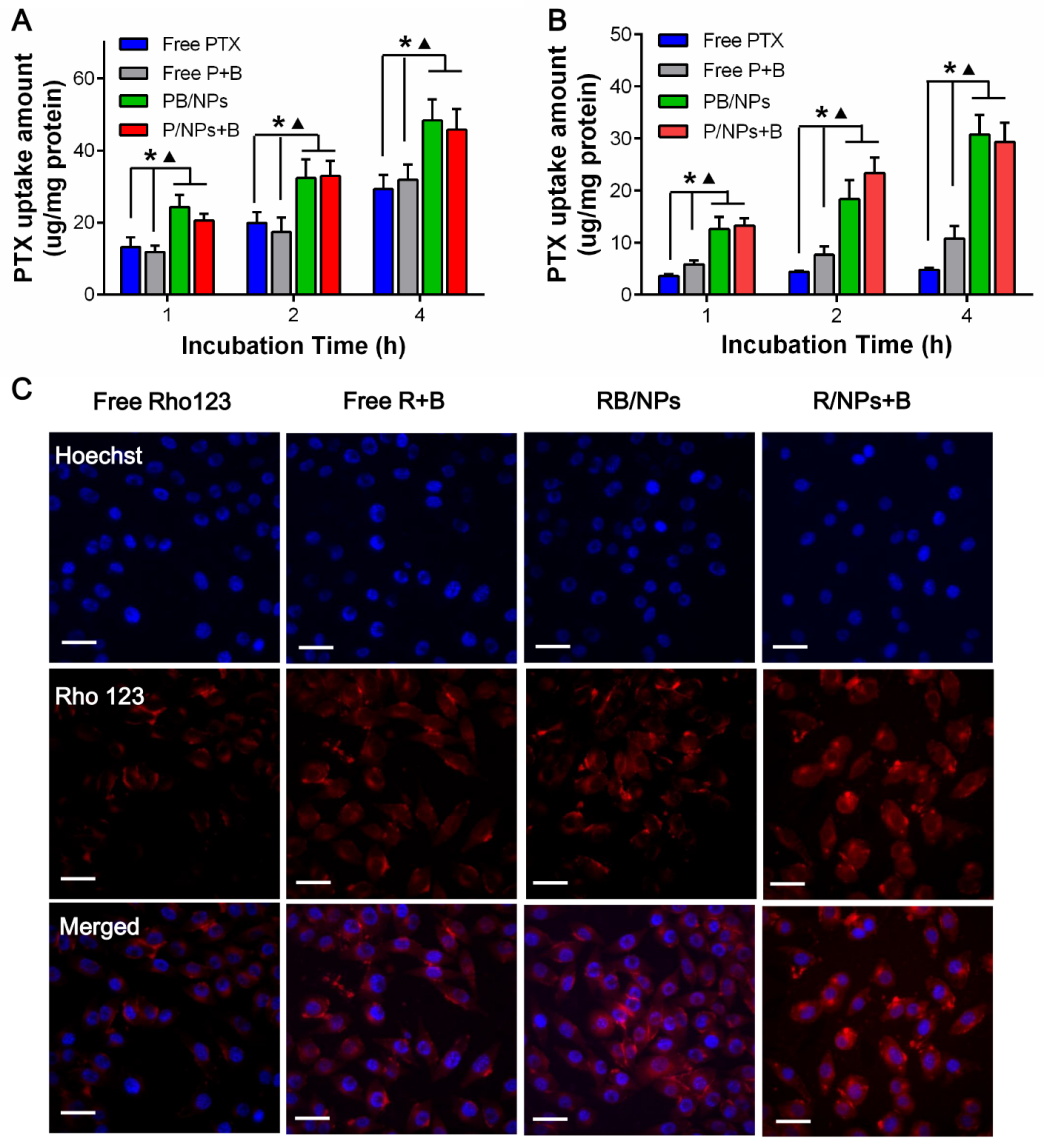
Intracellular PTX concentrations in A2780 cells **(A)** and A2780/PTX cells **(B)** after cells co-incubated with various PTX formulations for 1, 2, 4 h; **(C)** Fluorescent images of A2780/PTX cells incubated with Rho 123 formulations for 4 h. **Note**: *P<0.05, statistically significant differences with free PTX group; ^▲^P<0.05, statistically significant differences with free P+B group.

To evaluate that BNL and nano-carrier could enhance the intracellular PTX concentration in synergistically, various PTX samples were co-incubated with A2780 cells and A2780/PTX cells, respectively. We directly quantitated the intracellular PTX concentration on A2780s and A2780/PTX cells by HPLC. As shown in Figure 3A, PTX uptake in A2780 cells exhibited time-dependent manner. PTX loaded in NPs could significantly enhance the uptake capacity. However, the combination of BNL did not increase the intracellular PTX concentration in A2780 cells at all time-points, indicated that BNL barely could enhance PTX to entry into sensitive cancer cells. Nevertheless, because of the drug resistance, very small amount of free PTX could be entered into A2780/PTX cells during 4 h incubation (Figure 3B). The combination of BNL could remarkably increase PTX cellular uptake in A2780/PTX cells. Most importantly, PTX-loaded in NPs with both PB/NPs and P/NPs +B forms could significantly rise PTX uptake, in comparison with both free PTX and free P+B (P < 0.05). It would be contributed by easier conjugation and transmembrane transport resulted in nanoscale and slight positive potential of PEG-PAMAM NPs. Interestingly, PTX in the combination of BNL and PTX/NPs exhibited the similar cellular uptake, compared to that in PB/NPs, which might be for the reason that P-gp distributed in both inner and outer membrane of drug resistance cells. To visualize the uptake enhancement of BNL and PEG-PAMAM NPs in A2780/PTX, fluorescence probe Rho 123, as a P-gp substrate, was used to replace PTX. As shown Figure 3C, both (R+B)/NPs and R/NPs + B exhibited higher drug concentration in A2780/PTX, with the obvious red fluorescence in cytoplasm. Thus, the results indicated that both BNL and nano-carrier could increase PTX concentration in cells, leading to the much higher cellular uptake in A2780/PTX cells of PB/NPs.

### *In vitro* cytotoxicity

**Figure 4:**
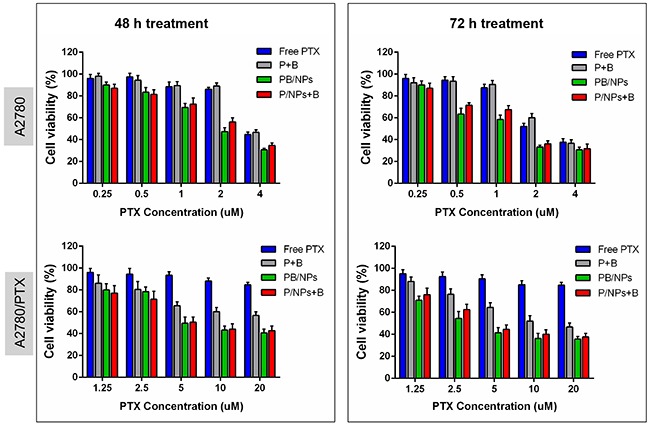
*In vitro* cytotoxicity of various PTX formulations of different equivalent PTX concentration in A2780/PTX cells for 48 h and 72 h

Figure 4 gave the clear evidence for the MDR reversal of BNL combination and PEG-PAMAM NPs introduction on drug resistant A2780/PTX cells. Primarily, after co-incubation of 72h, 100 μg/mL of PEG-PAMAM polymers induced 13.2% and 10.7% of total cell viability decrease on A2780 and A2780/PTX cell, respectively. This result indicated that blank PEG-PAMAM NPs possess high biocompatibility. As shown, compared to free PTX, free drugs combination, i.e. P+B, did not significant improve the cytotoxicity of PTX on the sensitive A2780 cells, instead of significantly enhancing that of PTX on drug resistant A2780/PTX cells. That would result from the reason that the combination of BNL could remarkably increase PTX concentration in A2780/PTX cells, while scarcely promote the cellular uptake of PTX in A2780 cells. Additionally, both PB/NPs and P/NPs+B exhibited higher cytotoxicity on these two cancer cell lines in comparison to P+B, with the time and dose-dependent manner. The encapsulation of PTX and BNL by PEG-PAMAM NPs could enhance the capacity to kill both A2780 and A2780/PTX cells. Specifically, after 72 h treatment, 20 μM of PTX cannot reach the IC_50_ values on A2780/PTX cells. Nevertheless, IC_50_ values of various PTX formulations were listed in Table 2 and Table 3. The enhanced cytotoxicity of PB/NPs and P/NPs+B was mainly attributed by the improvement of cellular internalization of NPs as noted in Figure 3. Additionally, although MDR reversal efficacy of PB/NPs seems higher than that of P/NPs+B on A2780/PTX cells, there was no significant difference between co-delivery PTX and BNL in one nanovehicle and the combination of the single PTX/NPs and free BNL. The phenomena could be explained by the reason that the over-expressed P-gp would distribute in both external surface and inner surface of cytomembrane. This result took a definite illustration for the synergistic effect of BNL combination and co-delivery by PEG-PAMAM NPs.

**Table 2:** The combination of BNL via NPs reverses PTX resistance in A2780/PTX cells after 48 h treatment

Formulations	A2780		A2780/PTX		
	IC_50_ value (μM)	FR	IC_50_ value (μM)	FR	Reversal index
PTX	3.73	1.00	84.35	22.61	--
P+B	3.86	1.03	25.57	6.85	3.30
P/NPs+B	2.35	0.63	8.38	2.25	10.04
PB/NPs	1.93	0.52	8.26	2.21	10.23

**FR: fold-resistance was calculated by dividing the IC_50_ value for PTX of A2780 and A2780/PTX cells in various PTX formulations by IC_50_ value for PTX of A2780 cells [25]**

**Table 3:** The combination of BNL via NPs reverses PTX resistance in A2780/PTX cells after 72 h treatment

Formulations	A2780		A2780/PTX		
	IC_50_ value (μM)	FR	IC_50_ value (μM)	FR	Reversal index
PTX	2.58	1.00	75.26	29.17	--
P+B	2.80	1.08	13.52	5.24	5.56
P/NPs+B	1.54	0.59	5.72	2.21	13.15
PB/NPs	1.26	0.48	4.10	1.58	18.46

**FR: fold-resistance was calculated by dividing the IC_50_ value for PTX of A2780 and A2780/PTX cells in various PTX formulations by IC_50_ value for PTX of A2780 cells [25]**

### Related mechanisms of MDR reversal

**Figure 5:**
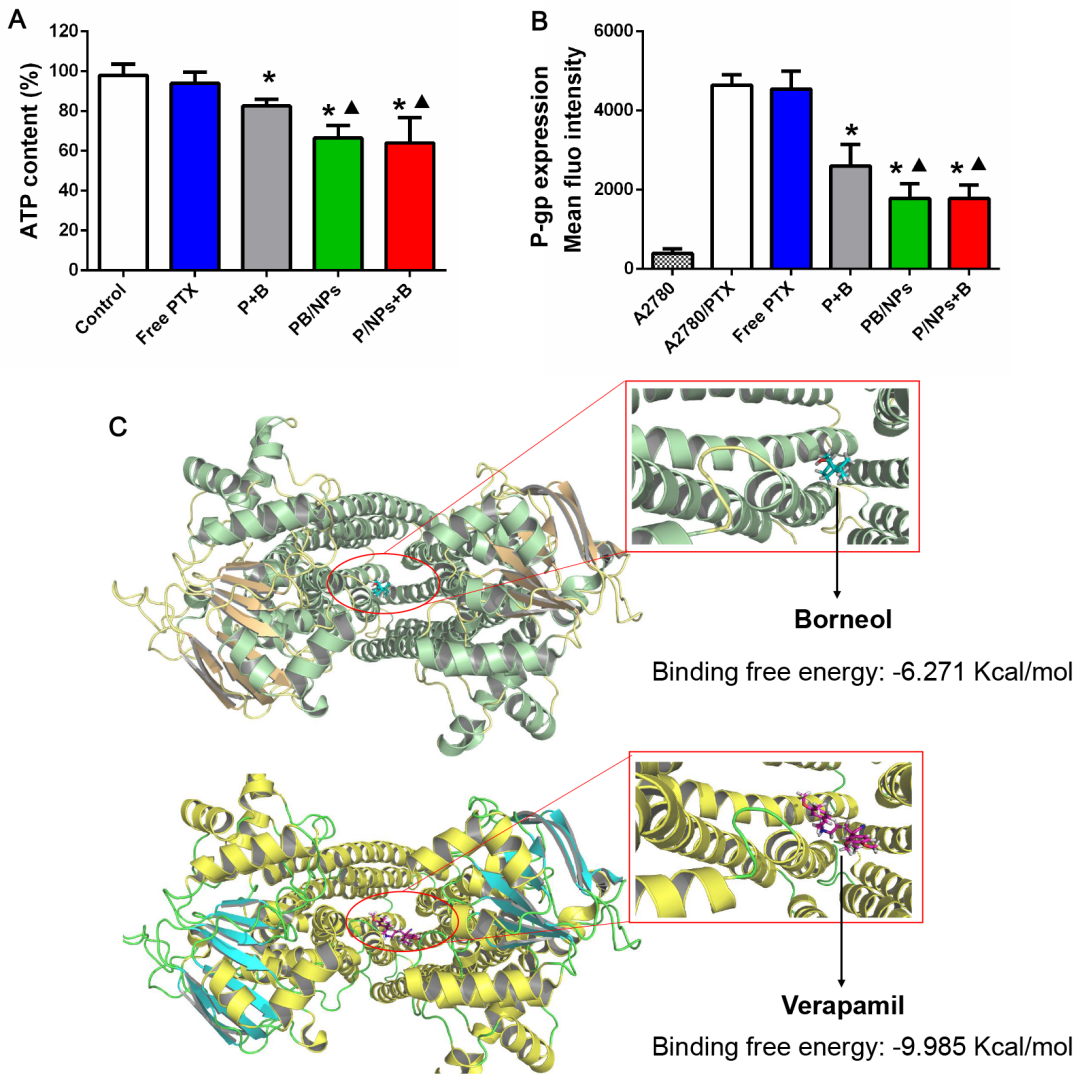
Inhibition on ATP level (A) and P-gp expression (B) of various PTX formulation with BNL combination in A2780/PTX cells; Docking interaction of BNL and verapamil with P-gp (C). Note: *P<0.05, statistically significant differences with free PTX group; ^▲^P<0.05, statistically significant differences with free P+B group.

After confirming the MDR reversal effect of the co-delivery NPs, we investigated the potential MDR reversal mechanisms. As well known, pumps of the ATP-binding cassette superfamily regulate the access of drugs to the intracellular space. So, we evaluated the influence of various PTX formulations on the intracellular ATP production in A2780/PTX cells. As shown in Figure 5A, bare decrease of intracellular ATP level was resulted, when cells were treated by single free PTX. However, cells with various formulations of PTX and BNL combination, including P+B, PB/NPs and P/NPs+B, showed a remarkably decreased ATP level. It indicated the importance to combine BNL with PTX. Moreover, mediated by PEG-PAMAM NPs, both PB/NPs and P/NPs+B showed higher inhibition efficacy on ATP production than free combination P+B. While, PB/NPs and P/NPs+B seem to exhibit the similar efficiency on ATP inhibition, partly because either BNL simultaneously loaded in NPs or PTX/NPs plus free BNL exhibited the equivalent efficacy to enhance cellular uptake. This data indicated that both co-delivery of PTX and BNL by PEG-PAMAM NPs and PTX/NPs plus free BNL would interfere with the mitochondrial function by inhibiting intracellular ATP production.

Although the fact that BNL could enhance intestinal absorption [26] and overcome blood brain barrier [11] which would be involved in P-gp activity inhibition has been reported, as we best known, to inhibit the overexpressed P-gp on drug resistant cancer cells was not yet reported. Herein, we employed the FITC labeled P-gp antibody to reveal the P-gp level on A2780/PTX cells with various PTX formulations treatment. As shown in Figure 5B, A2780/PTX cells exhibited much higher P-gp level than sensitive A2780 cells, indicating the overexpressed P-gp was one of the characteristics in drug resistant cells and this method could characterize P-gp level changes positively. Additionally, Figure 5A detailedly displayed that all PTX and BNL combination forms could significantly inhibit P-gp expression on A2780/PTX cells, providing the unequivocal evidence that BNL could markedly alleviate P-gp expression. Moreover, both co-delivery of PTX and BNL by PEG-PAMAM NPs and PTX/NPs plus free BNL exhibited higher P-gp inhibition efficacy than free PTX and BNL combination. This result confirmed that drugs encapsulation by NPs would benefit the P-gp inhibition, partly due to drugs could enter into cells much easier, mediated by the endocytosis of NPs.

To further reveal the mechanism of BNL binding to P-gp in molecular level, docking calculation was conducted. The residues of P-gp that interact with BNL and verapamil were shown in Figure 5C. BNL and verapamil were docked in P-gp molecule with the similar binding domain, in which particularly most binding sites of two drugs had a great deal of overlap. It suggested that both BNL and verapamil could recognize P-gp molecule by a common mechanism. However, verapamil (-9.985 kcal/mol) docked with higher free energy to the binding pocket in comparison to BNL (-6.271 kcal/mol), indicating the higher binding capacity. Even so, this result still demonstrated BNL was liable to bind with P-gp, and provided the evidence for its P-gp inhibition activity.

### MMP determination on A2780/PTX cells

**Figure 6:**
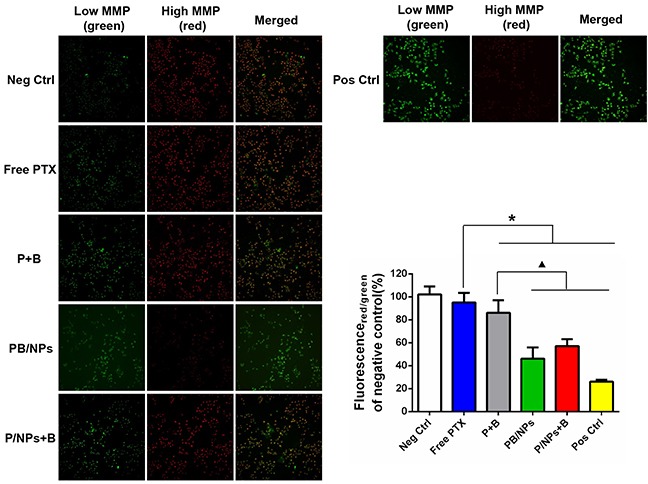
Inhibition effect of various PTX formulations on MMP of A2780/PTX cells by JC-1 detection kit with fluorescent image observation and quantitative determination by FCM **Note**: *P<0.05, statistically significant differences with free PTX group; ^▲^P<0.05, statistically significant differences with free P+B group.

The MMP of A2780/PTX cells treated by various PTX formulations was determined using JC-1, which undergoes a reversible transformation from a monomer (green florescence) into an aggregate form (red florescence) when it binds to a membrane with a high MP [27]. Mitochondrial depolarization (non-functional mitochondria) was indicated by a decrease in the ratio of the red/green fluorescence intensity. As shown in Figure 6, A2780/PTX cells with PBS and CPPP treatment as negative and positive control respectively showed obviously different, in which few red fluorescence was produced in negative control, instead of a mass of green fluorescence in positive control view. However, compared to free PTX, the formulations containing both BNL and PTX significantly decreased the average JC-1 red/green fluorescence intensity ratio, indicating that the combination with BNL could exert the enhanced mitochondrial depolarization effects. Moreover, compared to free P+B (red/green fluorescence intensity ratio at average 86.3%), both co-delivery of PTX and BNL by PEG-PAMAM NPs and PTX/NPs plus free BNL exhibited higher capacity to drop the ratio of the red/green fluorescence intensity, i.e. red/green fluorescence intensity ratio at 46.9% and 57.4% respectively, revealing the synergistic effect of nano-carrier loading and BNL combination on abating MMP and accordingly overcoming MDR.

### Apoptosis assay

**Figure 7:**
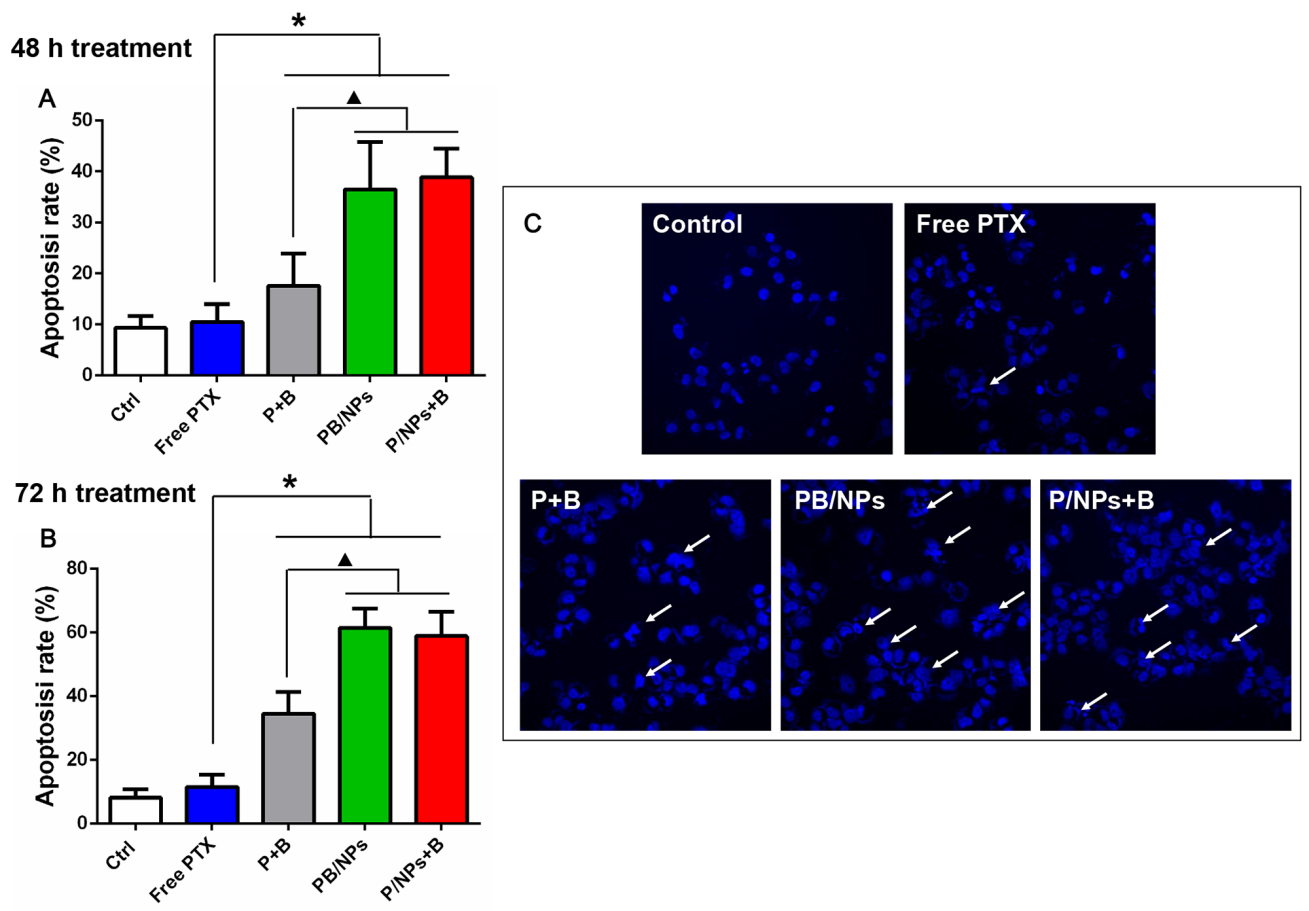
Apoptosis induction effect of various PTX formulations on A2780/PTX cells with 48 h **(A)** and 72 h **(B)** incubation by Annexin V-FITC/PI double staining method; Apoptosis nucleus observation of A2780/PTX cells treated by various PTX formulations with Hoechst 33342 staining. **Note**: *P<0.05, statistically significant differences with free PTX group; ^▲^P<0.05, statistically significant differences with free P+B group.

The pro-apoptosis effect of co-delivery of PTX and BNL on A2780/PTX cells was quantitatively investigated. Annexin V and PI dual staining method was employed to detect both early and late stages of apoptosis. Observing the results shown in Figure 7, it could demonstrate that the apoptotic cells increased obviously (early plus late apoptotic cells) in co-delivery of PTX and BNL groups, compared with negative control and free PTX group. As similar with the cytotoxicity result, various groups with PTX and BNL combination exhibited time-dependent manner in apoptosis induction. Particularly, both co-delivery of PTX and BNL by PEG-PAMAM NPs and PTX/NPs plus free BNL induced more apoptotic cells than free P+B. After 48h treatment, the total apoptosis rate induced by PB/NPs and P/NPs+B was 2.07 and 2.21 times more than that by free P+B. Similarly, the total apoptosis rate of PB/NPs and P/NPs+B was 1.77 and 1.62 times more than that by free P+B, after 72h treatment. These results indicated that PTX and BNL delivery by PEG-PAMAM NPs strengthened the induction apoptosis effect of dual-drug combination. The enhanced early and late apoptotic effect could been explained the enhanced cell endocytosis.

Moreover, the pro-apoptosis effect was also demonstrated by nucleus morphology observation. As shown in Figure 7C, both control group and free PTX group showed substantially regular and round nuclei under microscope view. Much more cells' nuclei with condensation and fragmentation were observed after exposed to P+B, PB/NPs and P/NPs+B for 48 h, which was recognized as characteristic of apoptotic cells. Thus, the result also suggested that co-delivery BNL and PTX with NPs could promote cell apoptosis.

### *In vivo* biodistribution

**Figure 8:**
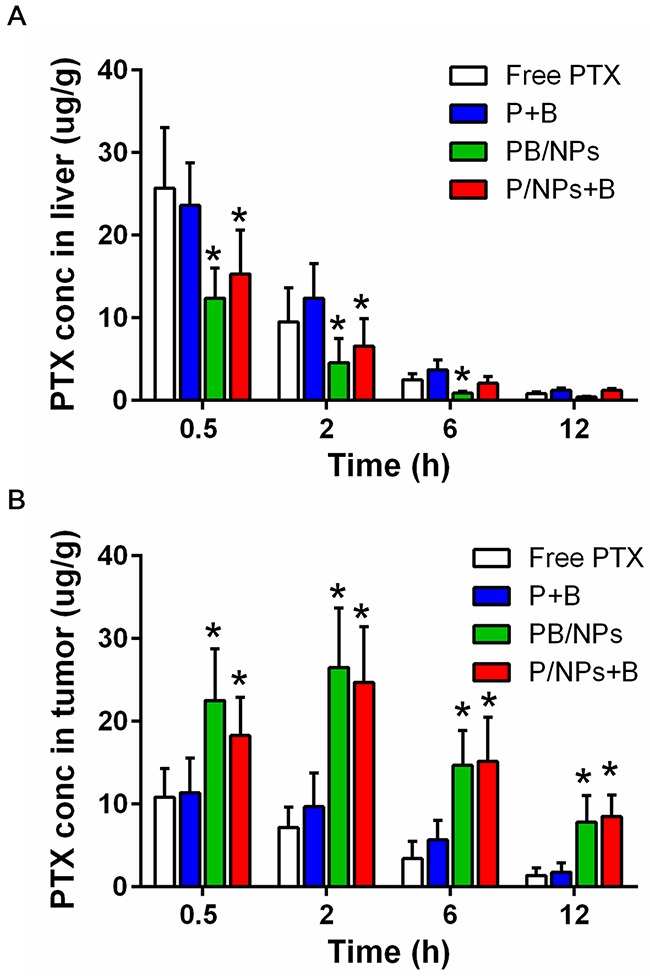
PTX bio-distribution of various PTX formulations in liver **(A)** and tumor tissues **(B)** of tumor-bearing mice at 0.5 h, 2 h, 6 h and 12 h post-injection. **Note**: *P<0.05, statistically significant differences with free PTX group

Based on the nano-sized effect for tumor accumulation and prolonged circulation of PEG chain, PEGylated NPs have been demonstrated to possess passive tumor targeting advantages. Herein, we evaluated whether drugs loaded in PEG-PAMAM NPs benefit to accumulation in tumor tissue. Moreover, liver is the primary organ in drug metabolism. More drugs remained in liver means fewer drugs accumulated in tumors. So, the concentration of PTX in liver and tumor after i.v. administration of various formulations containing 15 mg·kg^−1^ PTX were presented in Figure 8. As shown, with PTX concentration in liver gradually decreased during 12 h post-injection, PTX in tumors firstly climbed up and then declined. In detail, PTX in both free PTX and P+B with solution state displayed higher concentration in liver and lower accumulation in tumor at each time-point, compared to PTX loaded in NPs. It was clear in Figure 8B that PB/NPs and P/NPs+B exhibited much higher drug concentration in tumor tissue than both free PTX and P+B. For example, at 2 h post-injection, PTX accumulation in tumor exhibited the maximum value. Free PTX and P+B exhibited the similar drug accumulation. However, PB/NPs and P/NPs+B had 3.68-fold increase and 3.22-fold increase in comparison to free PTX in tumor drug concentration. All the above results concluded that PEG-PAMAM NPs could benefit to deliver PTX to the ovarian tumor tissue.

### *In vivo* antitumor efficacy

**Figure 9:**
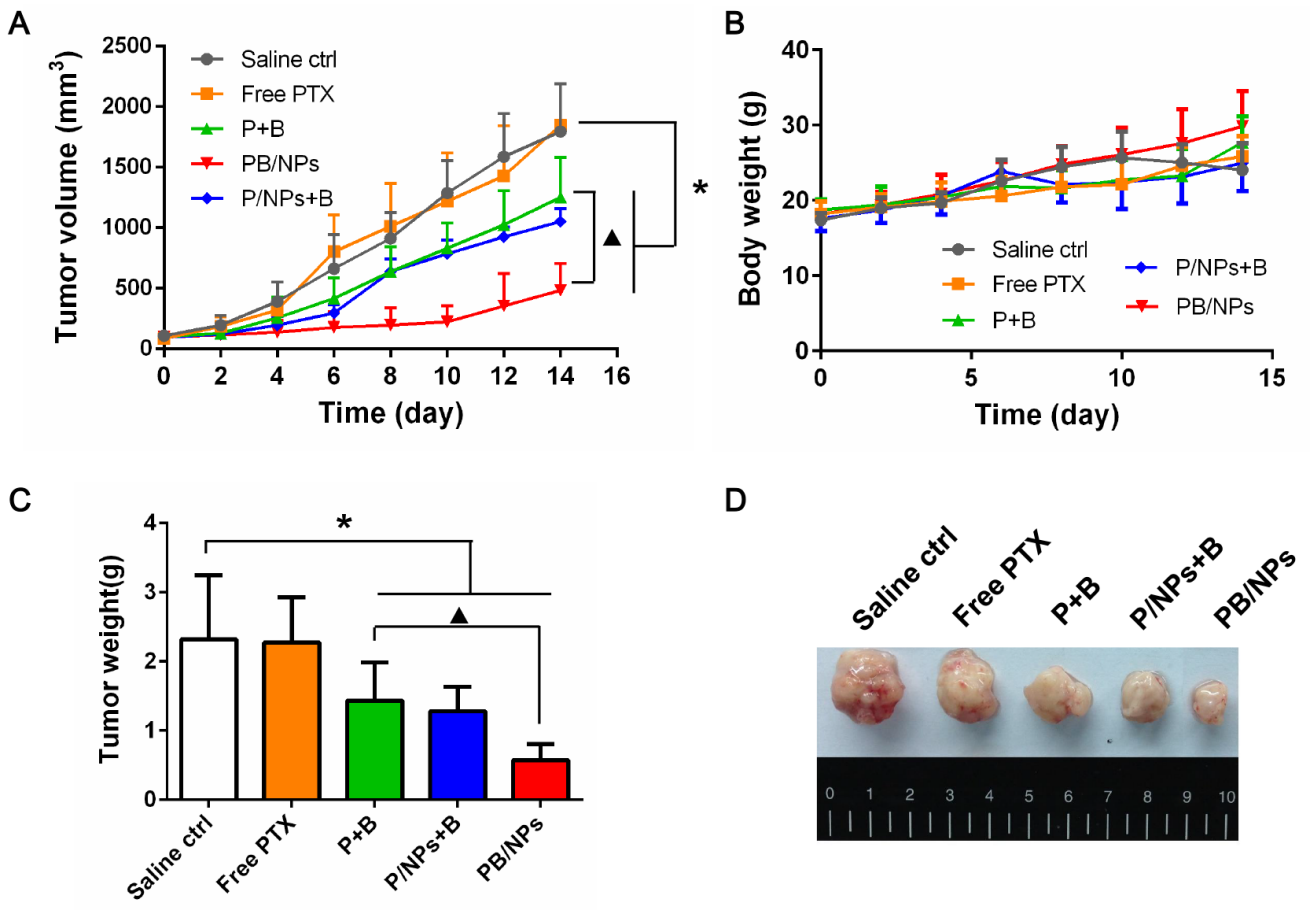
Tumor growth inhibition activities on tumor volume growth inhibition **(A)**, mice body weight **(B)**, and the collected tumor weight **(C)** of various PTX formulations in A2780/PTX tumor-bearing mice with 14 days administration. **(D)** Representative images of the collected tumors in each group.

Eventually, the therapeutic performance on drug resistant ovarian cancer of PTX and BNL co-delivery in PEG-PAMAM NPs was investigated using A2780/PTX tumor bearing nude mice. As similar with the scarce anticancer efficacy in MTT result, free PTX of 5 mg·kg^−1^ hardly can weaken the growth of drug resistant tumor. Mediated by BNL combination, P+B could slightly inhibit the continuous tumor progression in mice, which indicated that the combination with a P-gp inhibitor reliably could be beneficial to overcome MDR. Remarkably, tumor growth was drastically suppressed by PB/NPs (as shown in Figure 9A). Surprisingly, although P/NPs+B exhibited the similar anticancer efficacy as PB/NPs in above a series of *in vitro* tests, the inhibition effect on tumor volume growth of P/NPs+B was significantly lower than that of PB/NPs. The much lower antitumor effect of P/NPs+B would be led up to the different pharmacokinetic behaviors of free BNL and P/NPs in body circulation. Because of the physical mixture in P/NPs+B, BNL would be rapidly eliminated in body circulation, instead of the long retention time of P/NPs. The different pharmacokinetic behaviors of BNL and P/NPs certainly resulted in the inconsistent drug combination ratio of BNL and PTX in tumor tissue, and accordingly the limited MDR reversal effect. Nevertheless, PTX and BNL was co-loaded in PB/NPs, exhibiting the consistent pharmacokinetic behaviors owing to the NPs encapsulation. Thus, MDR reversal efficacy of PB/NPs was much higher than that of P/NPs+B, leading to the higher tumor growth inhibition activity. This result also indicated

**Figure 10:**
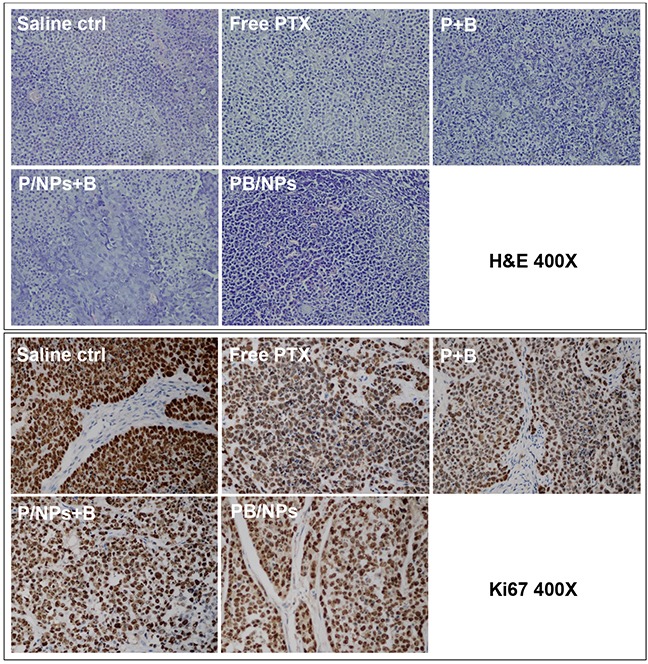
H&E stained section and Ki67 stained section of tumor tissues in various groups

The mice injected with P/NPs+B even manifested side-effect to a certain extent with trembling and body weight loss (Figure 9B). It would be for the reason that free BNL cannot keep as long circulation as P/NPs with a short half-life period less than 1 h. Although physical addition of BNL with P/NPs could inhibit P-gp function in cultured cells, the rapid elimination of BNL in body circulation resulted in the lower antitumor efficacy than PB/NPs. This result provided the clear witness for the advantage of multiple drugs delivery by nano-vehicles. Figure 9C and Figure 9D confirmed that mice following 14 day treatment with PB/NPs had the smallest tumor size. Notably, PB/NPs had no influence on mice body weight, indicated that PB/NPs had no significant systemic toxicity. In addition, the histological analyses by H&E images demonstrated that PB/NPs generated extensive tumor necrosis (Figure 10). Similarly, the fewest positive staining by Ki67 antibody in tumor section of mice treated by PB/NPs also confirmed that PB/NPs possessed the highest inhibition effect on cell proliferation in tumor tissue. Therefore, based on the synergistic effect of BNL combination and PEG-PAMAM NPs loading, PB/NPs showed better antitumor activity on A2780/PTX mice model and low systemic toxicity.

## MATERIALS AND METHODS

### Materials

Paclitaxel (PTX, purity ≥98%) and natural Borneol (BNL, purity ≥98%) were purchased from Melonepharma Co., Ltd. (Dalian, China) and National Institutes for Food and Drug Control. Polyamidoamine dendrimer G3 (PAMAM G3, Mw 6909) with 32 surface groups, N-hydroxysulfosuccinimide (NHS), 1-ethyl3-(3-dimethylaminopropyl) carbodiimide hydrochloride (EDC), 3-(4,5-Dimethylthiazol-2-yl)-2,5-diphenyltetrazolium bromide (MTT) and Hoechst 33342 were obtained from Sigma-Aldrich (St. Louis, MO, USA). Carboxylic acid functionalized poly(ethylene glycol) polymer (mPEG-COOH, Mw 2000) was purchased from Yare Biotech (Shanghai, China). All other chemicals were of analytical grade and were used as received.

The sensitive human ovarian carcinoma A2780 and paclitaxel-resistant A2780/PTX cell lines were obtained from KeyGen Biotech Co., Ltd. (Nanjing, China) and were maintained in DMEM (Life Technologies Co.) containing 10% fetal bovine serum (FBS, Gibco), 2 mM L-glutamine, penicillin-streptomycin solution (40 U/mL each, Gibco, Life Technologies Co.) at 37°C in a humidified CO_2_ (5%) incubator. Female BALB/c nude mice, aged 4-6 weeks (18∼22 g), were provided from Dashuo experimental animals Co., Ltd. (Chengdu, China) and maintained under specific pathogen-free conditions. Animals were kept under a 12 h light/dark cycle at the animal care facility, acclimatized for at least 7 days prior to the experiments and given a fresh diet with free access to water. All *in vivo* experiments were carried out under the guidelines approved by the Institutional Animal Care and Use Committee (IACUC) of Chengdu University of Traditional Chinese Medicine.

### Preparation and characterization of PEG-PAMAM NPs

#### Synthesis of PEG-PAMAM copolymer

Pegylated PAMAM G3 dendrimer was synthesized by the amidation reaction between carboxylic acid of PEG polymers and terminal amino groups of PAMAM dendrimer according to previous report [28]. Briefly, PAMAM G3 dendrimer, mPEG_2000_-COOH polymer, NHS and EDC with the molar ratio of 1:20:10:10 were dissolved in PBS with a few drops of trimethylamine. The mixture was steadily stirred with 600rpm under nitrogen atmosphere for 24 h. Subsequently, the mixture was dialyzed with dialysis tube (MWCO 5000 Da) against pH 7.4 PBS for 48 h to remove the residual PEG and NHS/EDC. PEG-PAMAM copolymers were obtained by lyophilization and characterized by ^1^H NMR.

#### Drug loading in PEG-PAMAM NPs

Due to the broad interior cavity of PAMAM dendrimer, liposoluble drugs could be easily loaded. PEG-PAMAM polymers were primarily dissolved in PBS pH 7.4 with 1.5 mg/mL. And then, 0.6 mg of PTX and 1.85 mg BNL in 2 mL of acetone: methanol (1:1) solution were added into 10mL of PEG-PAMAM solution drop by drop, and stirred for 4 h away from light. The residual organic solvents were removed in vacuum, and then the non-encapsulated, insoluble, hydrophobic drug was then removed by syringe filtration (0.22 μm filter). The single PTX NPs or blank NPs were prepared based on the similar process.

#### Characterization of PEG-PAMAM NPs

The particle size, polydispersity index and zeta potential of three batches of prepared NPs were determined by dynamic light scattering (DLS, Malvern Zetasizer Nano ZSP) at 25°C. The morphology and homogeneity of NPs was analyzed by a Tecnai G20 transmission electron microscope (TEM, FEI, Co., USA) at an operation voltage of 200 kV. To measure the encapsulation efficiency (EE) and loading efficiency (LE) of drugs, NPs were dissolved in methanol to disrupt the polymeric shells before analysis. Then, the PTX concentration was determined using a Waters e2695 HPLC equipped with a reverse phase C_18_ column (150×4.6 mm, 5 μm) at a maximum absorbance of 227 nm with acetonitrile/water (70/30, v/v) as the mobile phase at a flow rate of 1 mL/min. Additionally, BNL loaded was determined by a Gas Chromatography method with a capillary column (0.25mm×30m×0.25 μm). The EE and LE were calculated using the following equations, respectively:

$$\text{EE}(\%)=\frac{\text{amount of drug loaded}}{\text{amount of drug added}}\times 100\%$$

$$\text{LE}(\%)=\frac{\text{amount of drug loaded}}{\text{amount of drug loaded}+\text{polymer}}\times 100\%$$

#### *In vitro* release of loaded PTX

The release of PTX from PEG-PAMAM NPs was analyzed using dialysis. The free drug combination at an optimized ratio dissolved in a Cremophor EL and ethanol (1:1, v/v) mixed solvent was used as a control. Briefly, a 2 mL PTX/NPs suspension or free PTX mixture was transferred into a dialysis tube with MWCO 5,000 and was dialyzed against 40 mL phosphate buffer at pH 7.4 under stirring at 100 rpm. 1 mL of dialysis medium was sampled at predetermined periods (0.5, 1, 2, 4, 8, 12, 24, 48, 72, 96, 120, 144, 240 h) and determined by the aforementioned protocols. Meanwhile, 1 mL of fresh medium was added back to the dialysis medium after each sampling to maintain a constant volume. All drug release experiments were performed three times.

#### *In vitro* hemolysis assay

The hemolytic activity was evaluated with rabbit red blood cells according to literature [24]. Briefly, rabbit red blood cells (RBCs) were collected by fresh rabbit blood specimen centrifugation at 1000 rpm for 15 min, and diluted with 0.01M PBS to obtain a 2% RBCs suspension (v/v). PTX nano-scaled formulations, reconstituted from the prepared lyophilized micelles, PAMAM and PEG-PAMAM polymer were mixed with 0.5mL of RBCs suspension respectively. The blood compatibility test in PBS solution and in Triton X-100 solution (1%, v/v) were used as negative and positive controls, respectively. After incubation at 37°C for 2 h, the samples were centrifuged at 3000 rpm for 10 min; the supernatants were collected and analyzed for hemoglobin content by spectrophotometric detection at 570 nm. The percentage hemolysis (%) was calculated using the following equation:

Hemolysis (%)=(As‐An)/(Ap‐An)×100

where *As*, *An* and *Ap* are the absorbance of sample, negative and positive controls, respectively.

### Cellular uptake

The cellular uptake efficiency of free PTX, free P+B, P/NPs+B, and PB/NPs on drug sensitive A2780 and drug resistant A2780/PTX cells was measured by determining the intracellular drug concentration with HPLC. Briefly, cells were seeded in a 6-well plate at a density of 1×10^5^ per well for 24 h before the experiment. Cells were treated with different PTX formulations (equivalent PTX concentration of 2 μM) for predetermined time intervals (1, 2, and 4 h, respectively) at 37°C. At the end of the incubation period, cells were collected, washed three times with cold PBS and lysed with 1% Triton X-100. Both protein amount and PTX concentration in cell lysate were detected by BCA protein assay kit and HPLC analysis as previously reported Ref [29]. Cellular uptake of PTX was normalized with respect to the total protein content.

To visualize the intracellular localization of NPs on drug resistant A2780/PTX cells in aid of BNL, fluorescence probe Rhodamine 123 (Rho 123) was employed to replace PTX and loaded in NPs. Due to the lipophilicity, Rho 123 was loaded in PAMAM core by self-assembly. Specifically, Rho 123 with or without BNL, as well as PEG-PAMAM, dissolved in acetone were dropwise added into water phase, and steadily stirred for 4 h away from light. Rho 123 NPs was collected by filtration with 0.22 μm filter. And then, free Rho 123, combination of Rho 123 and BNL, R/NPs+B, and RB/NPs at an equivalent Rho 123 concentration of 1 μg/mL were co-incubated with 2×10^4^ A780/PTX cells/well. After 4 h, Cells were washed three times with cold PBS and fixed with 4% paraformaldehyde for 15 min. Hoechst 33342 was used to stain nucleus for 20 min. Fluorescence images were taken using a fluorescence microscopy (Olympus IX71, Tokyo, Japan).

### *In vitro* cytotoxicity

MTT assay was used to examine the *in vitro* cytotoxicity of different PTX formulations against A2780 and A2780/PTX cells. After seeded in a 96-well plate for 24 h, cells (5.0×10^3^/well) were treated with various samples for 48 h and 72 h. And then, cells were incubated with 1 mg/mL MTT medium for another 4 h. The solution was then removed and formazan crystals were dissolved with DMSO. The relative cell viability with untreated control was presented by detecting the 570 nm spectrophotometric absorbance with a microplate reader.

### Related mechanisms of MDR reversal

#### Intracellular ATP production determination

The intracellular ATP level was measured using an ATP assay kit (Beyotime, China) after A2780/PTX cells were treated with different PTX formulations. A2780/PTX cells at a density of 1×10^5^ cell/well were seeded and cultured in 12-well plates for 24 h, and were then incubated with different PTX formulations (5 μM PTX) for 8 h at 37°C. The content of ATP in the cells was determined as previous study [13].

#### P-gp activity measurement

P-gp expression in A2780/PTX cells treated by various PTX formulations was measured by using flow cytometry (FCM, BD, San Diego, CA, USA). Briefly, A2780/PTX cells (5.0×10^4^ cells/well) were seeded into 6-well plates separately and incubated for 24 h. After then, cells were treated by various PTX formulations with equivalent concentrations of PTX at 5 μM and BNL 100 μM for 4 h. At the end of incubation, 1 μL of P-gp antibody (FITC) was added and incubated for another 1 h. Collected by trypsin and washed by PBS, A2780/PTX cells were detected using FCM. The obtained values were expressed as folds of untreated controls.

#### Molecular docking study

In order to get a better understanding of the interaction mechanism of BNL with P-gp, molecular docking simulation based on X-ray crystal structure of recombinant mouse P-gp was carried out by AutoDock [30]. Verapamil was used as positive control. The 3D structure of the P-gp was downloaded from Protein Data Bank (PDB) (PDB ID: 3G60). P-gp protein was checked for any missing atoms and rectified by AutoDockTools 1.5.6. Charges were assigned using default parameters, then the final pdbqt file was created. The BNL/VMP were prepared by AutoDockTools 1.5.6. The center of grid box's co-ordinates was taken from the crystal ligand central atom and Affinity (grid) maps of 60×60×60 Å grid points and 0.375 Å spacing were generated using the Autogrid program. Docking simulations were performed using the Lamarckian genetic algorithm (LGA) to deal with the protein and inhibitors interactions. The docking parameters set to perform drugs docking experiment was derived from 100 different runs that were set to terminate after a maximum of 25 million energy evaluations, the number of individuals in population was set to 300.

### Mitochondrial membrane potential (MMP) measurement

A2780/PTX cells seeded at a density of 1×10^5^ cells/well were incubated with different PTX formulations at equivalent PTX concentrations of 5 μM. The cells incubated with serum-free DMEM culture medium were served as negative control. Carbonyl cyanide m-chlorophenylhydrazone (CCCP), a chemical inhibitor of oxidative phosphorylation, could cause rapid mitochondrial membrane depolarization as the negative control. After 24 h incubation, cells were washed, collected and then mixed in 0.5 mL culture medium and 0.5 mL pre-prepared JC-1 dyeing working solution following the manufacturer's protocol (Beyotime, China) by flow cytometry (BD, USA). Additionally, MMP changes resulted from PTX formulations were also visualized by fluorescence image assay [31]. After incubation, cells were washed two times with cold PBS and stained with JC-1 dyeing working solution following the manufacturer's protocol (Beyotime, China) for 30 min. Fluorescence images were taken using a fluorescence microscopy (Olympus IX71, Tokyo, Japan) with a 60× objective.

### Apoptosis assay

The apoptosis induction effect of PTX formulations in A2780/PTX cells was detected by Annexin V-FITC/propidium iodide (PI) assay. Briefly, cells were plated into 6-well plates with 2×10^5^ cells per well and treated for 48 h and 72 h with PTX formulations (equivalent concentration of 5 μM). Subsequently, cells were collected, washed with cold PBS, stained by 3 μL of Annexin V-FITC and 2 μL of 100 μg/mL PI successively, and re-suspended in 200 μL of binding buffer. After 15 min incubation, a total of at least 10,000 events were recorded by FCM.

Additionally, apoptosis in A2780/PTX cells was visualized by nuclear staining. Briefly, A2780/PTX cells were seeded in 96 well culture plates and treated with PTX formulations (equivalent concentration of 5 μM) for 48 h. After treatment, cells were stained with Hoechst 33342 at 37°C for 20 min in the dark, washed with PBS, and observed by fluorescence-inverted microscopy (IX73; Olympus, Tokyo, Japan).

### Biodistribution study *in vivo*

Biodistribution study was conducted on tumor bearing mice. To establish xenograft tumor models, A2780/PTX cells at a density of 5×10^6^ cell/mouse were subcutaneously injected into the upper back area of the nude mice. When the tumor volume reached approximately 1000 mm^3^, the mice were randomly divided into 4 groups with free access to food and water and treated with free PTX (dissolved in a 1:1 blend of Cremophor EL® and ethanol), free P+B, or P/NPs+BNL and PB/NPs with the equivalent PTX dose of 15 mg·kg^−1^ by tail vein injection. Then, at various time points (0.5, 2, 6, and 12 h) after dosing, mice were anesthetized and sacrificed. The tumor and liver tissues were harvested and preserved in a freezer for PTX extraction and determination. PTX determination in tissues was implemented by LC-MS.

### *In vivo* anti-tumor study

The *in vivo* anti-tumor studies were performed on female BALB/c nude mice bearing A2780/PTX cell xenografts. When the tumor volume reached approximately 200 mm^3^, the mice were randomly divided into 5 groups and treated with saline, free PTX (dissolved in a 1:1 blend of Cremophor EL® and ethanol), free PTX+BNL, or P/NPs+B and PB/NPs with the equivalent PTX and BNL dose of 5 mg·kg^−1^ and 40 mg·kg^−1^ by tail vein injection once every two days. The tumor volumes were measured every other day to assess the antitumor activities of the treatments. The body weights were measured simultaneously to indicate the systematic toxicities. After 7 times of administration, the mice were sacrificed and the weights of the tumor tissues were recorded. The tumor volume (mm^3^) was calculated by *V* = α · β^2^/2, where α and β represented the length and width of tumors. All of mice were sacrificed on day 15 and the tumor were histological analysis using the hematoxylin and eosin (H&E) staining and Ki67 antibody staining for immunohistochemistry.

### Statistical analysis

Results were given as mean ± standard deviation (S.D.). Statistical significance was tested by two-tailed Student's t-test or one-way ANOVA. Statistical significance was set at P < 0.05.

## CONCLUSION

In the present study, a dual-drugs co-delivery nano-sized system composed by the dendrimer-derivative PEG-PAMAM copolymer has been fabricated for PTX and BNL, a P-gp inhibitor, loading simultaneously. Based on the synergistic effect of PTX and BNL combination on MDR reversal by impairing drug efflux resulted from the over-expressed P-gp function, PEG-PAMAM NPs benefit to transport drugs into drug resistant A2780/PTX cells medicated by endocytosis. As expected, PTX and BNL co-delivery NPs exhibited higher cytotoxicity and apoptosis inducting activity on A2780/PTX cells *in vitro* and *in vivo*. Moreover, the advantage of multiple-drugs co-delivery also has been proved that PTX and BNL co-delivery NPs exhibited the improved tumor growth inhibition efficacy, in comparison to PTX NPs plus free BNL. To sum up, PEG-PAMAM NPs can be considered as a promising drug delivery candidate for efficient combination chemotherapy of PTX and BNL for MDR overcoming.

## References

[1] Ren F, Shen J, Shi H, Hornicek FJ, Kan Q, Duan Z Novel mechanisms and approaches to overcome multidrug resistance in the treatment of ovarian cancer. Biochim Biophys Acta.

[2] Lokadasan R, James FV, Narayanan G, Prabhakaran PK Targeted agents in epithelial ovarian cancer: review on emerging therapies and future developments. Ecancermedicalscience.

[3] Bao Y, Guo Y, Zhuang X, Li D, Cheng B, Tan S, Zhang Z D-alpha-tocopherol polyethylene glycol succinate-based redox-sensitive paclitaxel prodrug for overcoming multidrug resistance in cancer cells. Mol Pharm.

[4] Jabr-Milane LS, van Vlerken LE, Yadav S, Amiji MM Multi-functional nanocarriers to overcome tumor drug resistance. Cancer Treat Rev.

[5] Hamilton G, Rath B A short update on cancer chemoresistance. Wien Med Wochenschr.

[6] Niazi M, Zakeri-Milani P, Najafi Hajivar S, Soleymani Goloujeh M, Ghobakhlou N, Shahbazi Mojarrad J, Valizadeh H Nano-based strategies to overcome p-glycoprotein-mediated drug resistance. Expert Opin Drug Metab Toxicol.

[7] Liu Y, Fang J, Joo KI, Wong MK, Wang P Codelivery of chemotherapeutics via crosslinked multilamellar liposomal vesicles to overcome multidrug resistance in tumor. PloS One.

[8] Wang N, He T, Shen Y, Song L, Li L, Yang X, Li X, Pang M, Su W, Liu X, Wu Q, Gong C Paclitaxel and Tacrolimus Coencapsulated Polymeric Micelles That Enhance the Therapeutic Effect of Drug-Resistant Ovarian Cancer. ACS Appl Mater Interfaces.

[9] Wang S, Wang L, Chen M, Wang Y Gambogic acid sensitizes resistant breast cancer cells to doxorubicin through inhibiting P-glycoprotein and suppressing survivin expression. Chem Biol Interact.

[10] Chen Z, Gong X, Lu Y, Du S, Yang Z, Bai J, Li P, Wu H Enhancing effect of borneol and muscone on geniposide transport across the human nasal epithelial cell monolayer. PloS One.

[11] Fan X, Chai L, Zhang H, Wang Y, Zhang B, Gao X Borneol Depresses P-Glycoprotein Function by a NF-kappaB Signaling Mediated Mechanism in a Blood Brain Barrier in vitro Model. Int J Molec Sci.

[12] Chen J, Li L, Su J, Chen T Natural borneol enhances bisdemethoxycurcumin-induced cell cycle arrest in the G2/M phase through up-regulation of intracellular ROS in HepG2 cells. Food Funct.

[13] Assanhou AG, Li W, Zhang L, Xue L, Kong L, Sun H, Mo R, Zhang C Reversal of multidrug resistance by co-delivery of paclitaxel and lonidamine using a TPGS and hyaluronic acid dual-functionalized liposome for cancer treatment. Biomaterials.

[14] Meng J, Guo F, Xu H, Liang W, Wang C, Yang XD Combination Therapy using Co-encapsulated Resveratrol and Paclitaxel in Liposomes for Drug Resistance Reversal in Breast Cancer Cells in vivo. Sci Rep.

[15] Luong D, Kesharwani P, Deshmukh R, Mohd Amin MC, Gupta U, Greish K, Iyer AK PEGylated PAMAM dendrimers: Enhancing efficacy and mitigating toxicity for effective anticancer drug and gene delivery. Acta Biomater.

[16] Kesharwani P, Xie L, Banerjee S, Mao G, Padhye S, Sarkar FH, Iyer AK Hyaluronic acid-conjugated polyamidoamine dendrimers for targeted delivery of 3,4-difluorobenzylidene curcumin to CD44 overexpressing pancreatic cancer cells. Colloids Surf B Biointerfaces.

[17] Shcharbin D, Shakhbazau A, Bryszewska M Poly(amidoamine) dendrimer complexes as a platform for gene delivery. Expert Opin Drug Del.

[18] Yavuz B, Bozdag Pehlivan S, Sumer Bolu B, Nomak Sanyal R, Vural I, Unlu N Dexamethasone - PAMAM dendrimer conjugates for retinal delivery: preparation, characterization and in vivo evaluation. J Pharm Pharmacol.

[19] Zhong Q, Bielski ER, Rodrigues LS, Brown MR, Reineke JJ, da Rocha SR Conjugation to Poly(amidoamine) Dendrimers and Pulmonary Delivery Reduce Cardiac Accumulation and Enhance Antitumor Activity of Doxorubicin in Lung Metastasis. Mol Pharm.

[20] Gao Y, Gao G, He Y, Liu T, Qi R Recent advances of dendrimers in delivery of genes and drugs. Mini Rev Med Chem.

[21] Huang X, Wu Z, Gao W, Chen Q, Yu B Polyamidoamine dendrimers as potential drug carriers for enhanced aqueous solubility and oral bioavailability of silybin. Drug Dev Ind Pharm.

[22] Gurbuz MU, Ozturk K, Erturk AS, Yoyen-Ermis D, Esendagli G, Calis S, Tulu M Cytotoxicity and biodistribution studies on PEGylated EDA and PEG cored PAMAM dendrimers. J Biomater Sci Polym Ed.

[23] Jiang Y, Lv L, Shi H, Hua Y, Lv W, Wang X, Xin H, Xu Q PEGylated Polyamidoamine dendrimer conjugated with tumor homing peptide as a potential targeted delivery system for glioma. Colloids Surf B Biointerfaces.

[24] Zhang J, Han J, Zhang X, Jiang J, Xu M, Zhang D, Han J Polymeric nanoparticles based on chitooligosaccharide as drug carriers for co-delivery of all-trans-retinoic acid and paclitaxel. Carbohydr Polym.

[25] Muthusamy G, Balupillai A, Ramasamy K, Shanmugam M, Gunaseelan S, Mary B, Prasad NR Ferulic acid reverses ABCB1-mediated paclitaxel resistance in MDR cell lines. Eur J Pharmacol.

[26] He H, Shen Q, Li J Effects of borneol on the intestinal transport and absorption of two P-glycoprotein substrates in rats. Arch Pharmacol Res.

[27] Hong W, Shi H, Qiao M, Zhang Z, Yang W, Dong L, Xie F, Zhao C, Kang L pH-sensitive micelles for the intracellular co-delivery of curcumin and Pluronic L61 unimers for synergistic reversal effect of multidrug resistance. Sci Rep.

[28] Alibolandi M, Taghdisi SM, Ramezani P, Hosseini Shamili F, Farzad SA, Abnous K, Ramezani M Smart AS1411-aptamer conjugated pegylated PAMAM dendrimer for the superior delivery of camptothecin to colon adenocarcinoma in vitro and in vivo. Int J Pharm.

[29] Meng L, Xia X, Yang Y, Ye J, Dong W, Ma P, Jin Y, Liu Y Co-encapsulation of paclitaxel and baicalein in nanoemulsions to overcome multidrug resistance via oxidative stress augmentation and P-glycoprotein inhibition. Int J Pharm.

[30] Morris GM, Huey R, Lindstrom W, Sanner MF, Belew RK, Goodsell DS, Olson AJ AutoDock4 and AutoDockTools4: Automated docking with selective receptor flexibility. J Comput Chem.

[31] Chen F, Zhang J, Wang L, Wang Y, Chen M Tumor pH(e)-triggered charge-reversal and redox-responsive nanoparticles for docetaxel delivery in hepatocellular carcinoma treatment. Nanoscale.

